# The Link between SARS-CoV-2 Infection, Inflammation and Hypercoagulability-Impact of Hemorheologic Alterations on Cardiovascular Mortality

**DOI:** 10.3390/jcm10143015

**Published:** 2021-07-06

**Authors:** Johanna Sandor-Keri, Istvan Benedek, Stefania Polexa, Imre Benedek

**Affiliations:** 1 Clinic of Cardiology, University of Medicine, Pharmacy, Science, and Technology, George Emil Palade of Targu Mures, 540139 Targu Mures, Romania; polexa.stefania@gmail.com (S.P.); imrebenedek@yahoo.com (I.B.); 2 Clinic of Hematology and Bone Marrow Transplantation Unit, University of Medicine, Pharmacy, Science, and Technology, George Emil Palade of Targu Mures, 540139 Targu Mures, Romania; benedek.istvan@umfst.ro

**Keywords:** SARS-CoV-2, inflammation, hypercoagulability, myocardial infarction, hemorheology, PLR, NLR

## Abstract

The link between severe forms of severe acute respiratory syndrome coronavirus-2 (SARS-CoV-2) infection and cardiovascular diseases has been well documented by various studies that indicated a higher risk of cardiovascular complications in COVID-19 patients, in parallel with a higher risk of mortality in COVID-19 patients with underlying cardiovascular diseases. It seems that inflammation, which is a major pathophysiological substrate for both acute myocardial infarction and severe forms of COVID-19, may play a pivotal role in the interrelation between these two critical conditions, and hypercoagulability associated with SARS-CoV-2 infection could be responsible for acute cardiovascular complications. The neutrophil-to-lymphocyte ratio (NLR) and platelet-to-lymphocyte ratio (PLR) proved to be independent predictors for prognosis in acute coronary syndromes and systemic inflammatory diseases; therefore, they may be used as independent prognostic markers of disease severity in COVID-19 infection. The aim of this review is to present the most recent advances in understanding the complex link between SARS-CoV-2 infection, inflammation and alteration of blood coagulability and hemorheology, leading to major cardiovascular events.

## 1. Introduction

COVID-19 pandemic has caused a huge economic and health burden worldwide. The new coronavirus not only causes respiratory disease but also affects the cardiovascular system. There is growing evidence that COVID-19 infection is associated with severe cardiovascular events, such as acute heart failure, coronary artery thrombosis or myocardial infarction [1,2,3].

Different types of myocardial injury, acute heart failure, arrhythmia and venous thromboembolism appear to be more common among those infected with the new coronavirus. It has also been found that COVID-19-infected people are more likely to present with acute coronary syndrome (ACS), and the risk of heart failure has increased in a significant proportion of cases even before hospitalization for COVID-19 infection [2,4,5,6].

It is well known that in acute coronary syndromes, an acute inflammatory reaction superposed on a chronic inflammatory status may trigger an acute cardiovascular event. Inflammation-mediated endothelial dysfunction may lead to atheromatous plaque rupture and coronary thrombosis, leading to acute coronary syndrome and consequent severe myocardial injury.

Taking into consideration the strong inflammatory background of atherosclerosis, it is not surprising that cardiovascular complications, especially acute coronary syndromes, are more frequent during COVID-19 disease and a few weeks later. At the same time, COVID-19 patients frequently present an increase of D-dimers, suggesting that COVID infection is associated with increased systemic thrombogenicity [1].

However, there is still limited data in the literature about the neutrophil or platelet to lymphocyte ratios (PLR and NLR) in COVID-19 patients and their association with microvascular thrombosis. When elevated, these ratios indicate a higher risk for acute cardiovascular events and mortality, but their impact on cardiovascular mortality in COVID-19 patients is still under investigation [5]. The link between inflammation and thrombosis has been extensively studied in the latest years, but their complex association in COVID-19 patients, as well as their contribution to increased mortality in critical COVID-19 cases, is still a topic of great interest and not fully understood so far. Most data published in the literature focuses on the role of inflammation on severe COVID-19 cases, or on hypercoagulability in COVID-19 patients, which predisposes to cardiovascular complications, deep vein thrombosis and pulmonary embolism [4]. A clear description on the relationship between alteration of blood hemorheology, inflammation and cardiovascular complications in COVID-19 patients, integrating these different approaches in a common view, is still lacking in the literature.

The aim of this review is to present the most recent advances in understanding the complex link between SARS-CoV-2 infection and alteration of blood coagulability, underlining the usefulness of hemorheological parameters, especially PLR and NLR, leading to major cardiovascular events via an inflammatory-mediated mechanism. 

## 2. COVID-19, a Thromboinflammatory Disease

As indicated by recent data, inflammation caused by COVID-19 may be associated with alteration of coagulation parameters, leading to an increased risk of thromboembolic complications. It seems that a direct association exists between COVID-19 and blood hypercoagulability, since elevated levels of D-dimers and fibrin degradation products, as well as prolongation of prothrombin time, have been documented in severe forms of COVID-19 and are significantly associated with increased mortality [1,7,8,9,10,11].

The incidence of thromboembolic episodes in COVID-19 infection is relatively high. The pathomechanism of these severe complications involves COVID-19-specific pro-coagulants and a localized thromboinflammatory syndrome at the level of endothelial cells, which leads to a thrombotic disease that can affect all the systems in the human body [12].

Thromboembolic manifestations of COVID-19 may be divided in two categories: (1) arterial thrombotic complications, mainly coronary artery thrombosis, and (2) venous thrombotic complications, mainly deep vein thrombosis and pulmonary embolism.

Klok et al. reported that, despite low molecular weight heparin prophylaxis, arterial or venous thrombosis occur in 31% of COVID-19 cases hospitalized in intensive care units. In 81% of these cases the thrombotic event was pulmonary embolism, which occurred in 25 patients, while arterial thrombosis was recorded in 3.7% of cases [13]. In a study performed in Italy by Corrado et al. on 388 patients (16% from intensive care units), the rate of thrombotic events was 21%. Notably, 34.2% of the patients benefited from prophylactic anticoagulant treatment [14]. Stefanini et al. performed a study in 28 patients with COVID-19 infection and myocardial infarction. For 24 patients, myocardial infarction was the first manifestation of COVID-19, while the other 4 patients developed ST segment elevation myocardial infarction (STEMI) during hospitalization [15].

**Figure 1:**
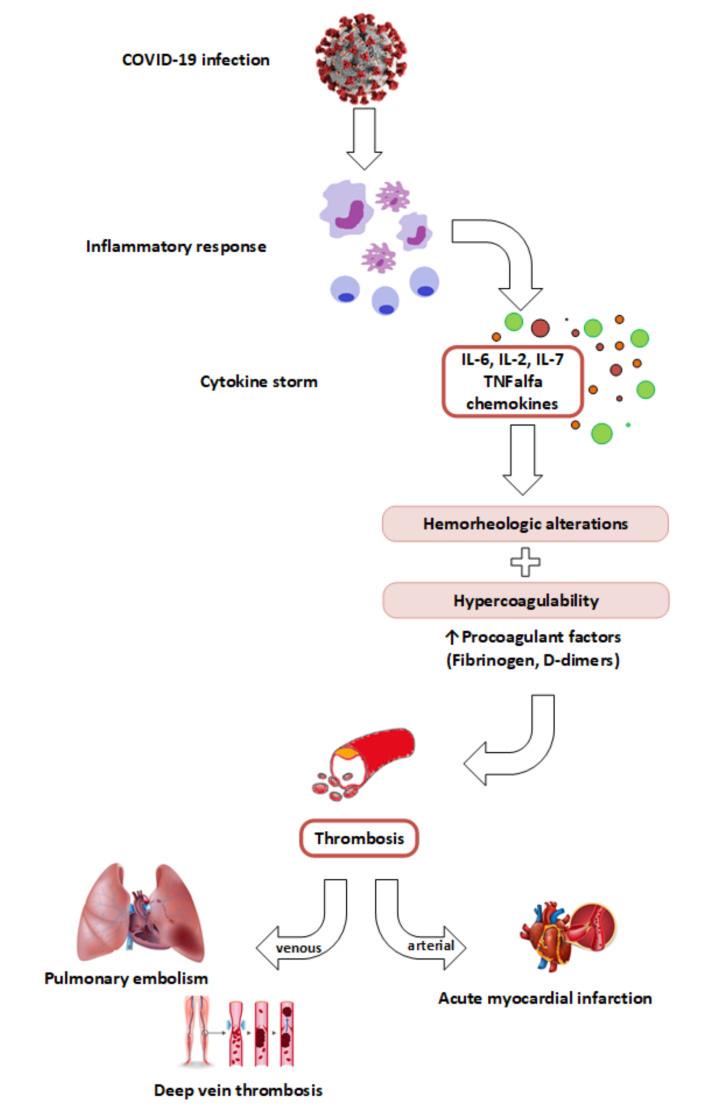
The link between SARS-CoV-2 infection, inflammation, cytokine storm and hypercoagulability leading to acute cardiovascular events (pulmonary embolism, myocardial infarction and deep vein thrombosis).

In the light of all these data, it becomes clear that a direct link exists between COVID-19 infection, inflammation and blood coagulability. Figure 1 illustrates the link between SARS-CoV-2 infection, inflammation and systemic hypercoagulability leading to acute cardiovascular events.

COVID-19 infection causes an overwhelmed inflammatory response with high levels of inflammatory cytokines, primarily tumor necrosis factor-α (TNF-α), interleukins (IL-2, IL-6, IL-7) and chemokines resulting in cytokine storm. This inflammatory reaction may trigger hemorhelogic alterations and blood hypercoagulability, predisposing to arterial and venous thrombosis. This may ultimately lead to severe cardiovascular complications, such as acute myocardial infarction, deep vein thrombosis and pulmonary embolism.

## 3. Platelet Activation and Acute Coronary Syndromes in COVID-19

The relation between platelet activation and myocardial infarction has been documented by various studies. Platelet activation is associated with a higher risk of irreversible damage of microcirculation despite successful recanalization, a condition known as “no reflow phenomenon”, which is favored by enhanced inflammation [16,17].

Several ratios between hemorheology parameters have been recently introduced as markers of increased risk associated with platelet activation or inflammation. For instance, PLR provides data on both aggregation pathways and inflammatory status. Therefore, this ratio is a better indicator of cardiovascular risk than the number of lymphocytes or platelets itself, since it reflects both inflammatory and coagulation pathways [18,19,20]. NLR is tightly connected with an increased level of inflammation in the body and may orient towards existence of an ongoing immune response in the human body [21,22,23,24,25].

**Figure 2:**
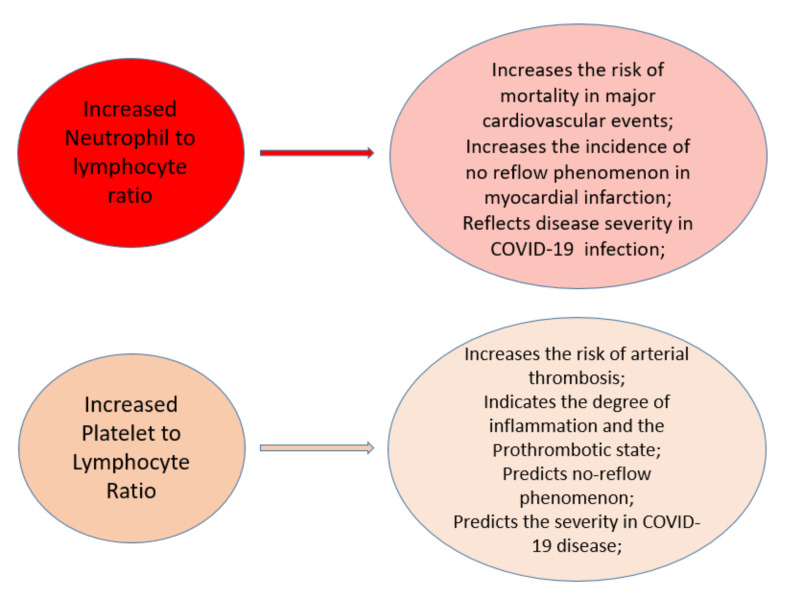
Hemorheologic indices (NLR, PLR) and their association with inflammation and thrombosis.

Figure 2 illustrates the link between different hemorheologic ratios, inflammation and coagulation favored by alteration of blood hemorheology.

NLR reflects the inflammatory milieu: increased NLR predicts the risk of major adverse cardiac events, the no reflow phenomenon in myocardial infarction and reflects disease severity in COVID-19 infection. PLR indicates the degree of inflammation and the prothrombotic state of the patient. An elevated platelet to lymphocyte ratio is associated with an increased risk of arterial thrombosis, predicts the no reflow phenomenon in myocardial infarction, and is associated with disease severity in COVID-19 patients.

A study conducted by Li et al. evaluated the prognostic value of PLR in elderly patients with acute myocardial infarction and found that PLR is an independent risk factor for negative outcomes, while NLR was the strongest predictor of adverse outcomes in stable and unstable coronary syndromes [26]. In a retrospective study, Yildiz et al. proved that high PLR and NLR are independent risk factors for development of the no reflow phenomenon in revascularized acute myocardial infarction [27].

Table 1 summarizes the most relevant studies published in the literature on the most commonly used hemorheological indices (PLR and NLR) and their relationship with acute cardiovascular events. All these data from the pre-COVID period indicate that hemorheology indices may serve as reliable predictors of clinical outcomes in the general population with acute coronary syndromes, COVID or non-COVID.

Particularly for COVID-19 patients, platelet activation, which may be triggered by systemic inflammation, is directly linked with the risk of arterial thrombosis, a major determinant of worse outcomes. Cardiovascular complications associated with increased aggregability in COVID patients include acute myocardial infarction and ischemic stroke, occurring with a high incidence in patients with activated platelets. At the same time, patients with pre-existing cardiovascular risk factors may have already established endothelial dysfunction, and therefore be exposed to a higher risk to develop severe forms of COVID-19 [28,29].

A study published this year on more than 300 consecutive patients presented to the emergency room showed that both PLR and NLR levels are elevated in COVID-19-positive patients, while absolute lymphocyte and platelet levels are elevated in COVID-19-negative patients compared to positive ones [37].

Moreover, a recent study on 131 patients diagnosed with COVID-19 in Wuhan explored whether the neutrophil-to-lymphocyte ratio (NLR) and platelet-to-lymphocyte ratio (PLR) are associated with the development of death in patients infected with SARS-CoV-2. This study found that an NLR of 3.338 may represent a cut-off for predicting all-cause mortality of COVID-19, while a cut-off of 2.3 may serve to indicate a potential risk for worse clinical evolution. However, PLR was not that useful for predicting clinical outcomes in COVID-19 population [38].

In a recent meta-analysis on 20 studies including 3508 COVID-19 patients, both NLR and PLR were strong predictors of clinical deterioration, patients with severe forms having significantly higher levels of NLR and PLR compared to non-severe cases (standard mean difference 2.8 (*p* < 0.0001) for NLR and 1.8 (*p* < 0.0001) for PLR [39].

All these data establish the role of hemorheology indices as independent prognostic markers of disease severity and risk of death in COVID-19 patients. However, it is still a matter of debate whether the problem is the ratio itself or the low number of lymphocytes. Since PLR reflects both inflammatory and aggregation status, it might seem difficult to identify the particular element associated with higher risk of cardiac events: the low number of lymphocytes or the high number of platelets [34]. The ratio between them may be especially relevant, since it reflects a particular disturbance of the hemorheologic balance, while aggregation and inflammation are strongly interconnected especially in critical cases. However, lymphocyte count is the common denominator in NLR and PLR, and elevation of both NLR and PLR is associated with increased mortality in COVID-19 infection. All these data indicate that decrease of lymphocyte count is directly involved in the complex pathophysiology of critical COVID-19 cases, ultimately leading to severe respiratory distress and death. Lymphocytes give rise to cytokines, which may be protective or disruptive. It seems that the diminished number of lymphocytes express a selective inability to generate protective cytokines such as type I interferon, and this may be related with increased COVID-19 mortality as well as with worse cardiovascular outcomes [38].

The fact that, prior to the COVID era, NLR and PLR have been described as strong predictors of no-reflow phenomenon and adverse events in non-COVID patients with acute myocardial infarction underlines that their role is not limited to inflammatory-mediated reactions in COVID-19. Other mechanisms linked to the complex inter-relation between inflammation and platelet aggregability, reflected by these ratios, are most likely involved in the unfavorable evolution of these cases [40].

## 4. The Link between SARS-CoV-2 Infection, Inflammatory Storm and Hypercoagulation

In 10–20% of patients infected with SARS-CoV-2, mainly in the elderly and those with co-morbidities, septic shock develops as a result of rapidly developing respiratory failure, or bacterial superinfection with high mortality [40,41].

Based on the clinical evolution, the time course of COVID-19 may be divided into three stages. Stage I is an early period of infection with variable respiratory or gastrointestinal symptoms or fever. Stage II is the lung phase when pneumonia appears. At this stage, antiviral therapy is usually necessary. Finally, phase III consists in systemic inflammation and cytokine storm with severe deterioration of the clinical status. The viral phase dominates in the first half of the disease and the inflammatory response of the immune system in the second half [42,43,44].

It appears that a decreased production of antiviral type I interferons (IFN-α/β) leads to a malfunction in viral protection of the organism. In addition, COVID-19 infection produces high levels of inflammatory cytokines, primarily tumor necrosis factor-α (TNF-α), interleukins (e.g., IL-1β, IL-2, IL-6, IL-7, IL-10, IL-12, IL-18, IL-33) and chemokines [45,46,47,48,49].

For instance, serum levels of IL-6, a prominent cytokine, are proportional to the severity of the infection and lymphopenia. Elevated plasma IL-6 levels were observed in three-quarters of severe COVID-19 patients and in only one-third of mild cases [50,51].

### 4.1. Cytokine Storm and Inflammation in COVID-19 Versus Sepsis

Cytokine storm is a critical condition that may occur not only in the evolution of COVID-19 but also in the evolution of sepsis. In sepsis, both pro-inflammatory and anti-inflammatory cytokines are elevated. Their role is mainly to eliminate the infection but, on the other hand, their excessive production can cause tissue and organ damage [52]. At the same time, cytokine storm represents the critical substrate for a lethal evolution in COVID-19 cases. However, the pathophysiologic mechanisms are quite different in these two conditions, since in COVID-19 cases the major problem is related to immunosuppression. 

The clinical syndrome of cytokine storm includes fever, low blood cell counts, liver and spleen enlargement, high ferritin, C-reactive protein (CRP), D-dimer and several cytokines (TNF-α, IL-2, IL-6, IL-7, G-CSF, chemokines). Elevated IL-6 and D-dimer serum levels have the highest predictive value for a severe outcome and the need for intensive care.

The proposed criteria for COVID-19-associated hyperinflammatory syndrome (cHIS) are: fever (>38 °C); macrophage activation (elevated ferritin levels > 700 µg/mL), hematological alterations (increased neutrophil to lymphocite ratio > 10, decreased hemoglobin concentration < 9.2 g/dL or low platelet count < 110.000/µL), coagulopathy (increase in D-dimer levels), increase in hepatic injury markers (lactate dehydrogenase or aspartat amino transferaze) and cytokinaemia (elevated IL-6 > 15 pg/mL, trigyliceride concentration > 150 mg/dL, C-reactive protein > 15 mg/dL) [45,46,47,53,54].

### 4.2. Cytokine Storm and Hypercoagulability

The main difference between sepsis and COVID-19 hypercoagulability is that while in sepsis systemic hypercoagulation and suppressed fibrinolysis leads to systemic coagulopathy, in COVID-19 a particular type of coagulopathy promotes local thrombus formation at different levels. It seems that venous thromboembolism and arterial thrombosis are more frequent associated with COVID-19 coagulopathy than with sepsis-induced coagulopathy [46,48].

Autopsies performed in patients who died of COVID-19 revealed direct invasion of endothelial cells by the virus. An important vasoconstriction appears in severe forms of SARS-CoV-2 infection, leading to organ ischaemia and inflammation accompanied by tissular edema, a procoagulant status with the consequential atherothrombotic complications. At the same time, SARS-COV-2-induced cytokine storm causes pulmonar vasculopathy secondary to severe endothelial dysfunction, which can result in microvascular thrombosis [48,49,55,56].

A correlation was found between D-dimer levels at admission and the risk of acute respiratory distress syndrome and death in COVID-19. A threshold of D-dimers ≥2 mg/L at any moment of hospitalization indicated the risk of death with a sensitivity of 92.3% and a specificity of 83.3% [56]. In addition, an increase in prothrombin time by >3 s and in activated partial thromboplastin time by >5 s were shown to represent independent predictors for thromboembolic complications [57].

### 4.3. Complement, Coagulation and Inflammation in COVID-19

**Figure 3:**
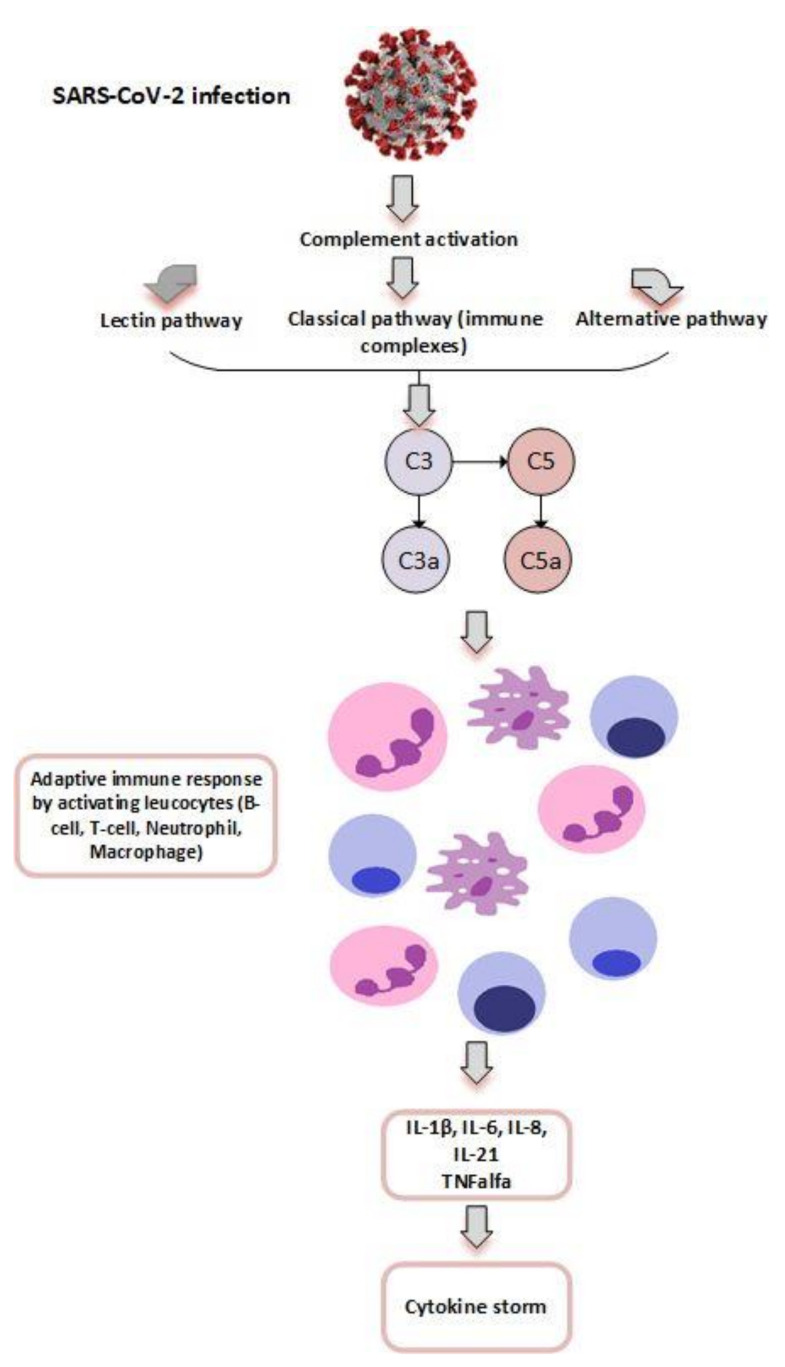
Effect of SARS-CoV-2 infection on complement activation.

One of the most important factors involved in the complex process of COVID-19 thrombosis is represented by complement activation. Complement may be activated via three major pathways: classical, lectin and alternative pathways, resulting in production of C3 and C5. Figure 3 illustrates the activation of C5a, a complement-activated product which triggers the adaptive immune response through the activation of various leucocytes (B and T lymphocytes, neutrophil granulocytes) [58]. When activated, complement helps to control bacterial and viral infection. However, in the context of severe COVID-19, excessive activation of the complement may become detrimental, favoring tissular damage and intravascular thrombosis. It has been well documented that complement activation plays an important role on cytokine and leukocyte activation, leading to neutrophil extracellular traps, which may promote thrombosis [59]. In SARS-CoV-2 infection, the viral spike protein binds to the ACE2 receptors, activating platelets, complement, cytokines and the coagulation cascade. At the same time, complement activates tissue factor, which further activates the coagulation cascade, linking SARS-CoV-2 infection with a highly thrombogenic status [58]. A recent study after severe acute respiratory syndrome coronavirus (SARS) and Middle East respiratory syndrome (MERS) epidemics demonstrated that C3-deficient mice expressed a less severe form of coronavirus infection, associated with higher degree of inflammation and thrombogenicity than the wild-type mice, indicating that complement activation is associated with a pro-thrombotic environment and may have a detrimental effect [60]. Therefore, a new therapeutic target in COVID-19 may be represented by inhibition of complement, with or without anti-inflammatory medication. Hertanto et al. described the beneficial effect of Non-SARS-CoV-2-specific intravenous immunoglobulin (IVIg) in COVID-19 through the modulation of inflammation, blocking the activation on innate immune effector cells, complement scavenging, and reciprocal regulation of T-cells, which leads to a decrease in plasma IL-6 and CRP levels [61].

Severe acute respiratory syndrome coronavirus-2 (SARS-CoV-2) activates the complement system which results in formation of the pro-inflammatory peptide (C3a) and the potent anaphylatoxin (C5a). This complement product activation leads to a cytokine storm which occurs within hours of infection due to elevation of inflammatory cytokines (Interleukin 1β, 6, 8, 21, Tumor necrosis factor-alpha). 

### 4.4. Hypercoagulability and Venous Thrombosis in COVID-19

Serum levels of D-dimers, a classical marker associated with increased blood coagulability, were elevated in more than 95% of COVID-19 patients admitted in intensive care units for acute respiratory distress syndrome and enrolled in a multicenter prospective cohort study runed by Helms et al. Moreover, 42.6% of them presented a thrombotic complication, which was pulmonary embolism in 16.7% of the cases [62].

In a prospective study of Wichmann et al., deep vein thrombosis was found during necropsy in 7 of 12 COVID-19 patients (58%), in whom no venous thromboembolism was suspected before death, and in 4 of these cases the direct cause of death was pulmonary embolism [63]. Another study published by Hippensteel et al. found out that 26% of critically ill patients hospitalized for COVID-19 have venous thromboembolism, located at the level of lower extremity deep veins, upper extremity veins, pulmonary veins or internal jugular vein [64].

Zhang et al. analyzed 143 patients hospitalized with COVID-19 infection in Wuhan, China and identified deep vein thrombosis during the observation period in 46.1% of them [65]. It has also been reported that more than 31% of COVID-19 infected patients admitted in the intensive care unit have a pulmonary embolism confirmed by X-ray or computed tomography scan [66].

Interestingly, it seems that venous thromboembolism and arterial thrombosis are more frequent associated with COVID-19 coagulopathy than with sepsis-induced coagulopathy [46,47]. It should be noted that the hypercoagulability associated with COVID-19 is different from the one recorded in sepsis, in the sense that in COVID-19, a particular type of coagulopathy promotes local thrombus formation at different local levels rather than at the systemic level. This particular type of coagulopathy leads to local thrombus formation at the site of pulmonary arteries and branches, which is a different mechanism than the one of pulmonary embolism resulting from embolization of a thrombus developed in a deep lower extremity vein, and explains the high rate of pulmonary embolism encountered in critical COVID patients. 

All these reports indicate a clear predisposition to increased blood coagulability leading to high rates of venous or pulmonary thrombosis in COVID-19 patients.

## 5. Biomarkers Associated with Worse Prognosis in COVID-19 Patients

Troponin elevation during COVID-19 infection can reveal an acute myocardial injury, which may be related to either myocarditis or acute myocardial infarction. Acute heart injuries were observed in 12% of COVID-19 patients and an increase of cardiac troponin, as the biomarker for myocardial injury, was reported in 5–25% of hospitalized patients for SARS-COV 2 infection [67,68,69].

A diagnosis of acute myocardial injury was established based on elevated cardiac biomarkers and electrocardiogram changes in 7–8% of COVID patients. The mechanisms responsible for acute coronary syndrome in SARS-COV 2 infection include arterial hypoxia, hypoperfusion, adrenergic stimulation, rupture of the atheroscerotic plaque, microthrombosis due to systemic inflammation and cytokine storm, spasm of the coronary arteries or angiotensin converting enzyme interceded injury [69]. A massive inflammation in any organ leads to release of cytokines such as IL-6, IL-8, TNF alpha which can trigger the inflammatory cells in the atherosclerotic plaque [70,71].

Yang et al. evaluated the potential risk factors for acute myocardial injury in patients infected with the novel coronavirus. They enrolled 149 COVID-19 positive patients and found that cardiac Troponin I levels were in significant correlation with ferritin levels, IL-6, IL-8 and high-sensitivity CRP [72].

Moriarty et al. demonstrated that an increase in serum levels of lipoprotein(a) in COVID-19 patients is associated with a higher risk for thrombosis. These lipoproteins are involved in the destabilization of the atherosclerotic plaques, which can be a reason for the apparition of an acute myocardial infarction [73].

While a large number of studies demonstrate the role of various biomarkers, including D-dimer, C-reactive protein, platelet count or hemorheologic indices for prediction of death, clinical deterioration or thrombotic events in COVID-19 patients, the data published so far do not sufficiently differentiate biomarkers associated with increased risk for arterial thrombosis from those associated with a higher risk of venous thrombosis. Although it might seem logical that biomarkers associated with increased risk of coronary events (such as C-reactive protein as a marker of persistent inflammation favoring coronary plaque progression) are related more to arterial than venous thrombosis, there is no clear evidence so far to document this hypothesis, which deserves further studies. 

## 6. Conclusions

COVID-19 is associated not only with systemic inflammation, but also with a pro-coagulant status which favors the development of severe cardiovascular events. The appearance of cytokine storm in the evolution of the infection triggers the sudden onset of a complex pathophysiological mechanism that leads to altered hemorheologic parameters and increased thrombogenicity. This may ultimately result in acute myocardial infarction or pulmonary embolism, making COVID-19 a severe thromboinflammatory disease. NLR and PLR, hemorheological parameters indirectly reflecting systemic inflammation, seem to be correlated with the severity of COVID-19 and especially with cardiovascular complications of this devastating disease.

## Figures and Tables

**Table 1:** Most relevant studies on hemorheological indices (platelet to lymphocyte-PLR and neutrophil to lymphocyte ratio-NLR) and their relationship with acute cardiovascular events.

Study	Data Collection Period	Patients	Age	Hemorheologic Ratio	Conclusions
Li et al. (2020) [26]	2012–2016	1001 patients with acute myocardial infarction (AMI) and primary percutaneous coronary intervention (PPCI)	441—49.7 ± 7.2 560—67.3 ± 5.6	PLR 165 ± 79 PLR 190 ± 107 *p* = 0.001	PLR is az independent predictor for aparition of adverse events during the hospitalization
Ayça et al. (2014) [28]	2010–2013	102 patients with stent thrombosis 450 patients with STEMI	54.6 + 11.1 58.3 + 7.4	NLR 7.00 + 5.77 4.60 + 3.87 *p* < 0.001	Higher NLR was associated with higher mortality rate in each group. Increased NLR can anticipate stent thrombosis and is associated with higher mortality rates in patients with STEMI.
Wahjuni et al. (2018) [29]	2012–2015	125 patients with acute coronary syndrome	*n* = 60, ≤45 years Gensini score > 53 (*n* = 23) Gensini score ≤ 53 (*n* = 37) *n* = 65, >45 years Gensini score > 53 (*n* = 36) Gensini score ≤ 53 (*n* = 29)	PLR 171.08 ± 83.54 88.51 ± 24.28 209.91 ± 164.45 133.01 ± 108.22	Optimum cut-off point for PLR was 111.06 for patients aged ≤45 years and 104.78 for patients aged >45 years
Mansiroglu et al. (2020) [30]	2015–2018	426 patients who undervent coronary angiography for acute coronary syndrome *n* = 102 unstable angina pectoris *n* = 223 non-STEMI *n* = 103 STEMI	64 ± 12 67 ± 12 67 ± 13	NLR < 0.001 2.92 ± 2.39 5.19 ± 4.80 7.93 ± 6.38	Statistically significant difference in the number of neutrophil counts and NLR between the types of acute coronary syndromes
Tamhane et al. (2008) [31]	1998–2004	2833 patients with ACS *n* = 564 STEMI *n* = 2269 non-STEMI	Low NLR *n* = 935 61 ± 13 years Medium NLR *n* = 948 65 ± 14 years High NLR *n* = 948 67 ± 13.8 years	NLR 1.82 (0.75) 3.56 (1.36) 9.10 (7.27)	NLR at admission can be successfully used for prediction of in-hospital and 6-month mortality
Yilmaz et al. (2015) [32]	No data available	251 patients with non-STEMI *n* = 82 without coronary thrombus *n* = 169 with coronary thrombus	60.68 ± 11.73 years 61.37 ± 12.34 years	NLR *p* < 0.001 3.17 + 1.52 4.12 + 1.89	Leukocyte count and NLR can be used to predict the presence of absence of a coronary thrombus.
Yildiz et al. (2014) [27]	No data available	287 patients with STEMI grouped by PLR *n* = 96 PLR: 88.2 (84.6–91.8) *n* = 96 PLR: 135.2 (132.0–138.4) *n* = 95 PLR: 231.7 (220.5–242.8)	57.6 + 13.3 years 60.2 + 13.7 years 64.5 + 13.2 years	NLR *p* < 0.001 3.44 + 1.47 5.24 + 2.20 8.44 + 3.83	Elevated PLR and NLR were indipendently and strongly associated with the no-reflow phenomenon in STEMI
Oylumlu et al. (2014) [33]	2012–2013	587 patients with acute coronary syndrome grouped by PLR *n* = 195 PLR: 83.9 ± 15.4 *n* = 196 PLR: 127.0 ± 13.8 *n* = 196 PLR: 214.0 ± 71.8	59.0 ± 12.2 61.7 ± 12.7 64.7 ± 13.7	NLR *p* < 0.001 2.50 (1.86–3.57) 4.11 (2.88–5.46) 7.04 (4.57–10.15)	An increased PLR can be an independent predictor of in-hospital mortality
Wagdy et al. (2016) [34]	2013	200 patients with STEMI grouped by the final TIMI flow *n* = 165-normal flow after PPCI *n* = 35–no reflow	No data available	NLR 5.44 ± 3.53 8.19 ± 3.05	NLR can predict the no-reflow phenomenon or in-hospital major advers cardiac event with 90.4% sensitivity and 51.5% specificity
Badran et al. (2020) [35]	2017–2019	200 patients with STEMI grouped by TIMI flow *n* = 58–TIMI 0-II *n* = 142–TIMI III	52.9 ± 11.1 years	PLR *p* = 0.001 199.4 ± 52 102 ± 53	Elevated pre-procedural PLR was predictive of the no-reflow phenomenon in STEMI
Wang et al. (2017) [36]	No data available	119 non-culprit plaques from 71 patients with ACS grouped by PLR assesed with optical coherence tomography *n* = 35 patients with 50 plaques (high) PLR > 109 *n* = 36 patients with 69 plaques (low) PLR < 109	59.77 ± 8.88 years 57.97 ± 10.51 years	Platelets, ×109/L 230.89 ± 45.47 202.75 ± 43.57	Increased PLR can be linked with vulnerable plaque features of non-culprit lesions
